# What format of treatment do patients with emotional disorders prefer and why? Implications for public mental health settings and policies

**DOI:** 10.1371/journal.pone.0218117

**Published:** 2019-06-10

**Authors:** Jorge Osma, Carlos Suso-Ribera, Óscar Peris-Baquero, Marta Gil-Lacruz, Luisa Pérez-Ayerra, Vanesa Ferreres-Galan, Mª Ángeles Torres-Alfosea, María López-Escriche, Olga Domínguez

**Affiliations:** 1 Departamento de psicología y sociología, Universidad de Zaragoza, Teruel, Spain; 2 Instituto de Investigaciones Sanitarias de Aragón, Zaragoza, Spain; 3 Departamento de psicología básica, clínica y psicobiología, Universitat Jaume I, Castellón, Spain; 4 CSM La Milagrosa, Pamplona, Spain; 5 Hospital Comarcal de Vinaròs, Castellón, Spain; 6 Hospital General Universitario de Alicante, Alicante, Spain; 7 CSM Sedaví, Valencia, Spain; 8 USM La Font de San Lluís, Valencia, Spain; Medical University of Vienna, AUSTRIA

## Abstract

**Objective:**

We analyzed the preference of three psychological intervention formats—individual, group, and online—in a sample of 267 patients with a primary diagnosis of emotional disorder in Spanish public mental health settings.

**Method:**

We studied patients’ preferences considering sociodemographic characteristics, diagnoses, history of psychological treatments, number of sessions, and satisfaction with past interventions.

**Results:**

Most participants (85.4%) preferred psychological treatment in an individual format, 14.2% in group, and 0.4% online. When comparing the people who chose individual and group treatment, no demographic or clinical differences were found. The arguments against group format were the lack of privacy and expression difficulties. Regarding online format, these included being considered impersonal and ineffective.

**Conclusion:**

The rejection of group and online psychotherapy formats allows us to define the actions we should carry out in public mental health settings to improve the acceptance of more cost-effective therapy formats.

## Introduction

Anxiety and mood disorders are the most prevalent emotional disorders (ED) in the general population [1]. ED refers to a group of disorders (e.g., anxiety, depression, somatoform, dissociative, and related disorders) that share a tendency to experience frequent and intense negative emotions, to present an aversive reaction to the emotional experience, and, subsequently, to engage in efforts to avoid the emotional experience [2]. In Spain, approximately two and a half million people are estimated to suffer from a depressive disorder, and almost two million people are thought to have a diagnosis of an anxiety disorder, which corresponds to 5.2% and 4.1% of the population, respectively [3]. In Spain, this results in a direct cost of 22,000 million euros (500 euros per capita and year) and an indirect expense of 78,000 million euros (1,300 annual euros per capita and year). The total sum of these expenses represents an alarming 2.2% of the Gross Domestic Product of the country [4]. The excessive demand for care in the Spanish public mental health system produces long waiting lists and makes it impossible to devote the necessary time to the treatment of all the people who request psychological care [4,5]. Consequently, it is necessary to invest more resources in the treatment of ED and, at the same time, to offer efficient and cost-effective treatments in the public system.

The literature has revealed the existence of various evidence-based techniques (e.g., exposure techniques for anxiety disorders or cognitive restructuring for depression) and therapies (Cognitive Behavior Therapy for anxiety and depression disorders) that have proven effective for the treatment of ED [6,7]. More recently, based on the identification of shared etiological and maintenance mechanisms among these disorders, Professor David H. Barlow and his team developed a single treatment protocol to address all these disorders. The unified protocol for the transdiagnostic treatment of ED [8] is an example of an intervention that might contribute to the reduction of the cost-benefit binomial in mental health. First, because it facilitates therapists’ training by using a single protocol for various disorders (all ED). Additionally, it can be applied to patients who present comorbidity, which makes it easy to offer group treatments when treatment candidates have different disorders. In fact, this approach has shown its efficacy both in individual [9,10] and group [11,12] formats. Our research team is carrying out a multicenter, randomized clinical trial in Spain to demonstrate the efficacy and cost-effectiveness of the unified protocol in a group intervention format for people with ED [13]. In the framework of this investigation, we noticed that a significant percentage of candidate patients refused to participate because of the group format. This made us wonder why some patients with ED were not keen on participating in a group intervention. The refusal of the group format has important implications for practice due to the elevated costs and dissemination limitations of individual treatments. In fact, several initiatives across the world have emphasized the need to reduce waiting lists [14] and improve the cost-benefit binomial of psychological treatments [2,15], but this is not likely to happen if individual treatments continue to be the mainstream. In the present study we investigate the preference of several psychological intervention formats (i.e., individual, group, or online) in a large sample of patients (n = 267) with a primary diagnosis of ED (or this being the most severe diagnosis) in Spanish public mental health settings, both in a quantitative and a qualitative manner.

Most studies on patient treatment preferences in the field of mental health have been interested in the preferences regarding the type of preferred psychotherapy (e.g., supportive, psychodynamic, or cognitive behavioral therapy) [16], type of treatment (e.g., psychotherapy versus pharmacotherapy) [17–19], type of specific techniques (e.g., live exposure versus exposure with virtual reality) [20], treatment adherence (e.g., Internet-Based and Face-to-Face Cognitive Behavioural Therapy) [21] or even the effect of initial provider type on treatment adequacy (e.g., mental health professional versus non-mental health professional) [22]. However, the study of format preferences (e.g., individual vs. online or individual vs. group), has not received the same scientific interest despite its aforementioned relevance.

Because our interest lies in the use of group and online therapy in public mental health contexts, we wanted to determine the arguments used by patients with ED to oppose to both formats in clinical settings in Spain. Exploring such barriers is important, as it might help reduce the resistance to group and online interventions. According to Piper [23], patients’ arguments against receiving group therapy include fears concerning the following aspects: loss of control, self-disclosure, criticism, rejection, and confidentiality issues, as well as a diminished sense of individuality, difficulties in understanding, and loss of privacy. Apparently, the benefits attributed to group therapy, such as the opportunity to learn from others (vicarious learning), receive feedback and support, or the fact that it is a more cost-effective format [24], are not strong enough arguments to change the negative attitudes of some patients towards group therapy. Actually, something similar happens in nonclinical contexts, where 62.9% of respondents would recommend individual therapy to a friend with serious problems compared to 33.3% who would recommend group therapy [25]. In this same work, 39.6% of respondents said that participating in a group treatment would generate greater discomfort than being in an individual intervention (17.1%).

Studies have also found that demographic characteristics influence format preferences. For instance, women are more willing to seek help in a group format (compared to an individual format) in the case of grief and depression. Also interestingly, the youngest and those with more previous psychotherapeutic experience valued group therapy more positively and evaluated it as being more useful [25].

Regarding patients’ preferences for online treatment, Klein and Cook [26] studied, in a sample of 218 Australian adults, whether differences existed between individuals who preferred online mental health services (77.1%) and those who preferred individual (face to face) services (22.9%). No differences were found between groups on demographic characteristics or on previous use of mental health services. Results indicated that preoccupation about the stigma of mental health was higher in respondents who preferred online services. As these authors mentioned, this is an important finding indicating that online mental health services could help to reduce the barriers for seeking care associated with mental health stigma. Focusing on the concerns about online services, more than half of the respondents in the aforementioned study expressed preoccupation about confidentiality of personal information. Other investigations have also described different arguments against online therapy, such as low perceived efficacy and the lack of information about the treatment process [27].

In sum, what the previous studies reveal is that the public image of group and online therapy is, in general, unfavorable. Contrary to group and online interventions, individual treatment appears to be well accepted, arguably due to the intimacy and privacy of this intervention format. However, arguments such as having a limited scope and being intrusive have also been proposed as barriers for the initiation of individual therapy [28]. It is unclear, however, whether these barriers in individual treatment only apply to individual therapy or all forms of psychological treatment.

The objectives of this study are, first, to explore the format preferences of patients with ED. Secondly, we intend to know the arguments they use to justify these preferences. Finally, we are interested in investigating whether sociodemographic variables, previous therapeutic experience and the satisfaction with it, and the type of diagnosis help us understand treatment preferences. By doing this, treatments could become personalized (e.g., by detecting if certain formats are preferred by certain groups of individuals) and, most importantly, barriers could be identified and addressed in the form of public campaigns (e.g., if certain groups of individuals oppose group format, they might have something in common, and plans to decrease barriers can be proposed) or pretreatment sessions. The information obtained in this study will allow us to define the actions that should be carried out by public health systems and clinicians to improve the acceptance of more cost-effective therapy formats, such as group and online therapy.

To the best of our knowledge, this will be the first study to investigate the preference of several psychological intervention formats (i.e., individual, group, or online) in a large sample of patients with a primary diagnosis of ED in Spanish public mental health settings. Our hypotheses are that: a) participants diagnosed with ED will prefer to receive psychological treatment in an individual format, then in a group format, and finally in an online format; b) the arguments against receiving group therapy will be related to the lack of privacy, confidentiality, and comfort; c) the arguments against receiving online therapy will be related to inefficacy, confidentiality, and lack of information about the treatment process; d) statistically significant differences will be found between people who prefer individual therapy and those who prefer the group and online format. Specifically, being a woman, being younger, having more years of psychotherapeutic experience and greater satisfaction with the therapy received, and having a diagnosis of depressive disorder will be associated with a more positive attitude towards group therapy.

## Methods

### Participants

The sample comprised 267 participants with a primary diagnosis of ED according to the *Diagnostic and Statistical Manual of Mental Disorders IV-TR* [29] and the *Diagnostic and Statistical Manual of Mental Disorders* (5th ed.; DSM-5 [1]) criteria. Both manuals are currently being used in the Spanish public mental health centers. Participants were aged between 18 and 76 years (75.3% women, mean age = 38.23, *SD* = 13.35). Table 1 presents the sociodemographic characteristics of the sample. Table 2 shows their clinical diagnoses.

**Table 1:** Sample characteristics (*N* = 267).

	*n*	*%*
**Marital status**		
Not in a relationship	141	52.8
Single	117	43.8
Divorced	23	8.6
Widowed	1	0.4
In a relationship	126	47.2
Married	92	34.5
In a relationship	34	12.7
**Educational level**		
Basic	156	58.4
Primary	50	18.7
Secondary	106	39.7
Higher	111	41.6
University	59	22.1
Higher	52	19.5
**Job status**		
Missing	11	4.1
Not working	128	47.9
Unemployed/no compensation	78	29.2
Unemployed/compensation	50	18.7
Working	128	47.9
Temporary	24	9.0
Part-time	27	10.1
Full-time	77	28.8
**Income (yearly)**		
Missing	103	38.6
< 16 thousand euros	78	29.2
>16 thousand euros	86	32.2

**Table 2:** Primary diagnoses.

	*n*	%
**Depressive Cluster**	48	18.0
Major depressive disorder	28	10.5
Dysthymia	12	4.5
Other specified depressive disorder	2	.7
Unspecified depressive disorder	6	2.2
**Anxious Cluster**	124	46.4
Panic disorder	25	9.4
Agoraphobia	12	4.5
Social Anxiety	6	2.2
Posttraumatic stress disorder	7	2.6
Obsessive-compulsive disorder	15	5.6
Generalized anxiety disorder	17	6.4
Other specified anxiety disorder	7	2.6
Unspecified anxiety disorder	35	13.1
**Mixed Cluster**	95	35.6
Adjustment disorder	88	33.0
Somatoform disorder	7	2.6
**Total**	267	100.0

### Procedure

The study sample was recruited by clinical psychologists who collaborated in the multicenter study on the efficacy of the unified protocol for the transdiagnostic treatment of ED in Spain [13]. Specifically, the sample was recruited at the CSM La Milagrosa (Pamplona), Hospital Comarcal de Vinaròs (Castellón), Hospital General Universitario de Alicante (Alicante), CSM Sedaví (Valencia), and USM La Font de San Lluís (Valencia). Clinical psychologists participating in the study include licensed psychologists with between 8 and 20 years of experience in clinical assessment and delivering of CBT interventions, as well as clinical psychology residents with 2 to 4 years of experience in clinical psychology. Clinical psychologists invited participants to fill the paper survey (see the Instruments section below) after they fulfilled the inclusion criteria and before they were enrolled in the randomized control trial. In the trial, patients could either be assigned to a group or an individual format, but the assessment of preferences was prior to assignment. Participants who refused to participate in the trial before or after the randomization were offered the treatment as usual in their mental health centers. However, the exact number of participants who refused the assigned condition is not available because a number of clinicians failed to collect this information.

The inclusion criteria to participate in the study were: 1) Principal diagnosis (most interfering and severe) of an ED (anxiety disorder, mood disorder, adjustment disorder, or related disorders); 2) The patient is over 18 years of age; 3) The patient is fluent in Spanish. The exclusion criteria were: 1) The patient presents a severe mental disorder (bipolar disorder, schizophrenia, or an organic mental disorder); 2) Suicide risk is present at the time of assessment; 3) If there is previous history of an addiction to substances, the patient has used the substance in the last three months.

The study and its procedures were approved by the ethics committee of all collaborating centers and all participants signed a written informed consent prior to participation.

### Assessment

*Anxiety Disorders Interview Schedule Lifetime Version for DSM-IV* (ADIS-IV-L;[30]; translated into Spanish by Botella & Ballester, [31]). The ADIS-IV-L is a semistructured interview designed to assess anxiety, mood, somatoform, and substance use disorders according to the criteria of the *DSM-IV* [29]. The ADIS-IV-L was administered by clinical psychologists participating in the study, as described in the Procedure section. Test-retest reliability varies, depending on the study, from .68 to 1.00. A reliability analysis was not conducted in the study due to time constraints in Spanish public health settings.

*Patient's psychological format preferences survey* (*ad hoc*). The survey requested information on the patients’ preferred format (“In case of needing a psychological treatment, in what way would you prefer to receive it?”). Participants had to choose between individual, group, or online. The participants rated the preferred format with 1 (*first place*), 2 (*second place*), 3 (*third place*), or No answer (*I would never choose it*). The use of forced-choice, rank orders was motivated by research showing increased validity of these formats compared to rating scales [32]. Next, the reasons for choosing a format in the first place were investigated by means of three free response questions, each referred to one of the three formats (“Why did you choose that format in the first option?”). We also asked participants about their last choice (“Why did you choose that format in the third place?”) and the opposed format (“Why would you never choose this format?”). In addition to format preferences and their justification, we included sociodemographic items and information about their history of psychological treatments, average number of sessions received in case of having previously received psychological treatment, and satisfaction with the intervention format received in the past. Satisfaction with previous treatment was explored using an 11-point Likert scale ranging from 0 (*very low satisfaction*) to 10 (*very high satisfaction*).

### Statistical analysis

Descriptive analyses were performed, including sociodemographics and preferences reported by participants. Then, the sociodemographics and previous treatment characteristics of patients who preferred individual and group format were compared. Differences in mean scores were calculated using Student's *t-*tests when variables were quantitative (age, number of previous therapy sessions, and degree of satisfaction with previous therapy), while a Chi-square test was conducted for qualitative variables (sex, educational level, marital status, employment status, income, and diagnosis). Such differences could not be calculated for online format because only one participant chose online format as the first choice. We also investigated demographic and preference differences across recruitment centers. A Bonferroni correction was used to minimize the risk of type I errors due multiple comparisons, so a more restrictive alpha of .001 was set. All analyses were carried out using the statistical program SPSS version 23 [33].

Regarding the qualitative analysis of the free response questions to their justification to select/oppose to a format, we analyzed the answers by means of an open coding approach [34] conducted by 2 researchers (JO and OP). The answers to each question were grouped on the basis of shared thematic features, resulting in a reduced number of ideas.

## Results

With respect to format preferences, 85.4% of participants preferred the individual format (*n* = 228), 14.2% the group format (*n* = 38), and only 0.4% the online format (*n* = 1). We omitted the participant who selected the online treatment in the following analyses due to the low frequency of this format. We then explored whether people who preferred individual treatment and those who chose group delivery differed in any of the study variables (age, sex, educational level, marital status, employment status, income, number of psychological therapy sessions they had attended in the past, satisfaction with the past intervention format, and type of ED). As shown in Tables 1 and 2, we grouped the response options of some of these variables to reduce the number of cells in the analyses so that we had enough observations in each cell (e.g., marital status was reduced from five to two categories: in/without a relationship).

Regarding the history of previous psychological treatment, the results indicated that 61.1% of the participants (*n* = 124) had previously received treatment compared to 38.9% (*n* = 79) who had not. The average number of sessions for those who had received treatment in the past was approximately 15 sessions (*SD* = 18.31). Even though the satisfaction rates among participants who had received individual therapy (*M* = 7.04, *SD* = 2.80) and group therapy (*M* = 7.08, *SD* = 2.43) were similar, the results indicated that individual intervention was clearly preferred (Table 3).

**Table 3:** Preference order according to intervention formats (*N* = 267).

		Format	
	Individual	Group	Online
**First place**	228	38	1
**Second place**	38	174	24
**Third place**	0	14	100
**Would never choose it**	1	41	142

When comparing the people who chose individual and group treatment, no demographic and clinical differences were revealed according to our more restrictive alpha level. Specifically, age (*t* = -2.6, *p* = .011), sex (*χ*^2^ = 0.9, *p* = .351), educational level (*χ*^2^ = 1.2, *p* = .264), marital status (*χ*^2^ = 2.0, *p* = .160), job status (*χ*^2^ = 1.2, *p* = .280), income (*χ*^2^ = 0.4, *p* = .523), satisfaction with previous individual therapy (*t* = 0.7, *p* = .512), satisfaction with previous group therapy (*t* = -0.5, *p* = .603), the number of previous therapy sessions (*t* = -1.1, *p* = .290), and the type of diagnosis (*χ*^2^ = 0.2, *p* = .902) were comparable irrespective of their actual format preference.

Next, we explored a number of relevant demographic and preference differences across recruitment centers. According to the corrected alpha value (*α* = .001), centers were comparable in terms of age (*F* = 3.4, *p* = .005), sex distribution (*χ*^2^ = 4.8, *p* = .446), type of diagnosis distribution (*χ*^2^ = 19.4, *p* = .036), and preferred format (*χ*^2^ = 12.3, *p* = .031).

Table 4 shows the participant's arguments to choose individual, group, or online therapy format, as well as the arguments to refuse those formats. In summary, participant's most frequent argument to choose individual format was that it "facilitates expressing problems" (*n* = 54). No arguments were reported to refuse individual therapy, but some patients (*n* = 4) would prefer other formats because they experienced “difficulties with interpersonal communication”. Participants’ most frequent argument to choose group therapy was that this format facilitates "sharing experiences" (*n* = 12). A considerable number of participants (*n* = 22) indicated that they would only choose group therapy as a third choice because they "preferred other treatments". Three participants opposed to receive group format because of the "lack of privacy" (*n* = 2) and their "difficulties in expressing problems in public" (*n* = 1). Finally, just one participant selected the online format as a first choice, arguing that this form of intervention was "convenient" for him. Arguments against online format included being too "impersonal" (*n* = 43) and "inefficient" (*n* = 23).

**Table 4:** Arguments for and against treatment format preferences.

Treatment format	Arguments to choose it as first choice	Arguments to choose it as third choice	Arguments to reject it
Individual	Facilitates expressing problems (*n* = 54); Privacy/Anonymity (*n* = 50); Personalized attention (*n* = 40); Sense of closeness/Comfort (*n* = 36); Efficacy (*n* = 17); Previous experience (*n* = 11)	Previous experience (*n* = 4); Difficulties in interpersonal communication (*n* = 4); Lack of motivation (*n* = 1); Cannot share experiences with others (*n* = 1)	
Total (*n*)	*n* = 208	*n* = 10	*n* = 0
Group	Share experiences (*n* = 12); Efficacy (*n* = 1); Previous experience (*n* = 1);Novelty (*n* = 1)	Difficulty expressing problems in public (*n* = 7); Preference for other treatments (*n* = 22); Lack of closeness (*n* = 5) No previous experience (*n* = 6); Low efficacy (*n* = 8); Lack of privacy (*n* = 9) Rejection of group therapy (*n* = 5)	Lack of privacy (*n* = 2); Difficulty expressing problems in public (*n* = 1)
Total (*n*)	*n* = 15	*n* = 62	*n* = 3
Online	Comfortable (*n* = 1)	Impersonal (*n* = 51); Ineffective (*n* = 27); Dislike (*n* = 14); Only as a complement (*n* = 5)	Impersonal (*n* = 43); Inefficient (*n* = 23); Dislike (*n* = 20); Uncertain (*n* = 9)
Total (*n*)	*n* = 1	*n* = 97	*n* = 95

## Discussion

In this study, we intended to explore the format preferences of patients with ED attending the public mental health system in Spain. Overall, we found that the majority of participants preferred to receive psychological treatment in an individual format, followed by group format, and, rarely, in an online format. The results are consistent with what was proposed in our hypotheses and also with previous research showing the preference of most people for individual therapy compared to other treatment options [24,28,35]. Our study also evidences a high rejection rate of online therapy which, as reflected by our qualitative analysis, could be explained by the lack of familiarity with this format. In fact, unlike other countries [36,37], online therapy for the evaluation and treatment of psychological disorders is not offered in the public health system in Spain.

It is remarkable that the order of preference was not influenced by prior experience with group therapy and patients’ satisfaction with previous treatment. These findings indicate that previous experience with group therapy, despite being positive, does not change the patients’ preference in favor of the group format and individual treatment remains the first choice. Something similar was evidenced when a group of primary care users were asked about their preferences between psychological or pharmacological treatment. While most people preferred psychological treatment, pharmacological treatment has been the most widely used by people who have required treatment [38]. Despite this remains speculative at this stage, it is possible that results are explained by the social stigma associated with psychotherapy in general [39] and group therapy in particular [25,39].

The present study results revealed that no demographic differences explained treatment preferences when using a restrictive alpha level to control for multiple comparisons. These results do not support our hypothesis nor do they coincide with findings obtained by other studies where being female and young were associated with a more positive attitude towards psychotherapy in general [27,40] and group interventions in particular [25,38,40]. In another study, being female was related to the preference for individual therapy [28]. Apparently, it would important to account for gender differences when considering treatment format preferences, but our findings do not support this idea. Perhaps the contradiction comes from the sample characteristics because the studies mentioned include nonclinical participants. It is possible that the fact that participants in the present study were patients with a clinical diagnosis seeking psychological treatment explains the differences with other studies where patients did not have a clinical diagnosis.

One of the most important goals of this study was to know the pros and cons of the formats of psychological treatment offered. The patients in our study offered the following arguments in favor of individual therapy: the ease of expression, intimacy/privacy, and personalized attention. These results regarding intimacy/privacy were also found in the study of Shechtman and Abeer Kiezel [28]. The findings indicate that, if given the change to choose, people with ED prefer the dyadic relationship with a therapist, appreciating the intimacy and privacy of this interaction. In this same work, participants proposed arguments for not choosing individual therapy, of which being limited in scope and intrusiveness were the most frequent barriers. In our study, none of the patients expressed arguments against individual therapy. These results were expected, as this format is offered regularly and frequently in the Spanish public mental health system. Even though the agreement between patients’ expectations and what is offered to them is positive in terms of treatment adherence and satisfaction [35,41,42], it can also become an obstacle for the acceptance of formats that improve the cost-benefit binomial in mental health.

The most frequent reason for choosing group therapy as a first alternative was the possibility of sharing experiences. The opportunity to learn from others and to receive feedback and support is probably related to this argument [25,28]. The strongest arguments against group therapy included the lack of privacy, poor perceived efficacy, the perception that the relationship with the therapist is less close in a group format, and the difficulty that some individuals experience in expressing themselves in front of others. Only the lack of privacy was also identified in previous studies, so the new arguments revealed in the present investigation should also be taken into consideration when addressing the acceptance of group format [23,28].

The present study results revealed a majority of patients with ED who, when given the possibility to choose, would prefer individual therapy. In addition, a large number of patients were reluctant to participate into group therapy because they perceived that this format does not guarantee privacy, that is, that group therapy will involve sharing information about their personal and intimate life that they are not willing to reveal to other patients. While it is true that, in a group context, communicating personal information is encouraged, the amount of detail and description given depends on the individual. In addition, this process of sharing information occurs over time and occurs in a context of support among peers, without judgments and maintaining confidentiality. Regarding the lack of efficacy, patients should be informed about the accumulated data on the effectiveness of group therapy for the treatment of ED [11,12,43]. Finally, people who have more difficulties in social skills, such as communicating or expressing their thoughts and feelings in social contexts (e.g., group therapy), may refuse this format and feel more comfortable in a more private interaction. Some therapy formats may be more appropriate for some patients than others, but, in our clinical experience, we have seen how, over time, shy people are able to participate and interact more frequently, which gives them greater self-confidence and personal satisfaction.

It would therefore be advisable for therapists to discuss these issues with patients who are advised to receive group therapy before choosing the treatment format [44]. This idea is indicated by Piper [23] when proposing the concept of pretherapy training which, among other possibilities, can include the therapists providing information and discussing with the patients the aspects of group therapy that they are concerned about, in this case, the lack of privacy, effectiveness, and difficulties of expression. Through this simple and inexpensive process, it would be possible to adapt patients' expectations and to increase acceptance of group therapy in public mental health centers. More complex initiatives such as public awareness campaigns might also be helpful but will require the involvement of entities.

The only argument in favor of online therapy has been its convenience, while the arguments to choose it as the last option and also to reject it have been its consideration as being ineffective and impersonal. These counterarguments have not obtained empirical support; in fact, the article of Soto-Pérez et al. [37] contains various studies that show the satisfaction of patients treated online and the non-existence of differences in the therapeutic alliance among those treated online and those treated face-to-face. In addition, the effectiveness of online therapy is currently very well established [37,45,46]. As mentioned previously, the pretherapy training solution could be useful also for the improvement of the acceptance of online therapy if the Spanish public mental health system was to consider its use.

### Limitations

In relation to the limitations of the study, we first want to note that, despite the large sample used, it would have been desirable to have a larger sample so to increase the robustness of the results. Another shortcoming is that, due to time constraints in public mental health settings in Spain, we couldn't conduct a reliability diagnosis study, which is desirable in clinical control trials, and we were not able to explore comorbidity diagnoses, so only main diagnoses are available. We could have included culture/ethnicity in demographic data, given that this construct has been shown to be relevant in the study of preferences of individual versus group therapy [28]. Additionally, while the assigned condition did not influence the study results on preferences (the information on preferences was collected prior to assignment), it would have been interesting to collect data on the number of participants who refused their assigned condition (i.e., group or individual).We could also have examined the opinion of the therapists regarding the formats of psychological intervention they prefer, their arguments, degree of confidence, and training, etc., which would have greatly enriched the study. The addition of the patient’s opinion about each format individually using a Likert scale as opposed to the study of the preference in a forced-choice ranked format would also have provided additional interesting information for cost-benefit analyses. Also importantly, it should be noted that previous exposure was more frequent for individual treatment, which might partly explain the preference for this format due to familiarity or previous positive experience with therapists. While we intended to control for the effect of previous exposure to each treatment format on preference by including this in the analyses, a design that sampled exposure evenly would provide more robust findings. However, it is important to note that this sample is representative of the people with a diagnosis of ED who are attended to in Spanish public mental health units and preferences were comparable across centers, which should make the results relevant in applied settings.

## Conclusions

This work shows that only a small percentage of patients with a diagnosis of ED would agree to receive group therapy, and that virtually all of them would refuse online therapy. The rejection of these two forms of intervention has important implications in the management of public mental health services, especially when it is necessary to reduce waiting lists and to improve the cost-benefit binomial of psychological treatments.

The negative attitudes of patients toward group and online formats are based on the lack of privacy, effectiveness, and the difficulty of expression regarding the former, and the consideration of the latter as impersonal and ineffective. As proposed by Piper [23], pretherapy training could be an appropriate solution to this problem. According to this perspective, therapists should generate a script that includes the benefits of these formats, relevant information about their characteristics and their functioning, as well as their short-term disadvantages (e.g., embarrassment, insecurity, coldness), so that these issues can be discussed with the patients.

In the study, we have evaluated a number of demographic and clinical factors and included quantitative and qualitative data in an attempt to understand patients’ preferences for each format. However, the cost-free nature of individual psychological treatments in Spanish public health settings might also the patients’ reduced interest on more cost-effective treatments, such as group or online interventions. Therefore, the changes we hope will occur in the psychological support of patients who come to the public health services (i.e., an increase in the selection of more cost-effective alternatives) will require that mental health professionals and administrators are convinced of the efficacy, effectiveness, and efficiency of group and online therapy, and as a result, they invest in training and in the services and tools that are necessary for their proper implementation.

## Supporting information

S1 File Treatment preferences survey. Original version
(DOCX)

S2 File Treatment preferences survey. English version
(DOCX)
