# Supplementary material for: What format of treatment do patients with emotional disorders prefer and why? Implications for public mental health settings and policies
Source: PLoS One. 2019 Jun 10;14(6):e0218117. doi: 10.1371/journal.pone.0218117 (PMC6557569; doi:10.1371/journal.pone.0218117)
Supplement: S1 File — Original version (DOCX) [file pone.0218117.s001.docx]

**Cuestionario sobre las preferencias de formato de intervención para tratamientos psicológicos**

(Jorge Osma, 2016. Universidad de Zaragoza)

**Centro:**

**Diagnóstico principal:**

**Código identificador del usuario: Edad: Sexo:**

| Primarios |  |
| --- | --- |
| Secundarios (Bachiller, FP, etc.) |  |
| Universitarios |  |
| Postgrado/Máster/Doctorado |  |

**Estado civil: Nivel de estudios:**

| Soltero/a |  |
| --- | --- |
| Casado/a |  |
| En pareja |  |
| Divorciado/a |  |
| Viudo/a |  |

**Situación laboral: Nivel de ingresos familiar al año:**

| Sin trabajo ni paro |  |
| --- | --- |
| En el paro |  |
| Trabajo temporal (o menor a la media jornada) |  |
| Trabajo media jornada |  |
| Trabajo jornada completa |  |

| 0-16.000 euros |  |
| --- | --- |
| 16.001-17.500 euros |  |
| 17.501-19.000 euros |  |
| 19.001-21.000 euros |  |
| 21.001- 24.000 euros |  |
| 24.001-66.451 euros |  |

| **Sí** |  | **No** |  |
| --- | --- | --- | --- |

**¿Ha recibido tratamiento psicológico previamente?** (Da igual que haya sido ininterrumpidamente o en distintos periodos de vida)

**Si ha respondido SÍ en la pregunta anterior, por favor responda a las siguientes preguntas.**

**¿Cuál fue el número de sesiones aproximadas que recibió?** (por ejemplo, 13 sesiones).

|  |
| --- |

**Por favor, indique el formato en el que recibió el/los tratamientos y su grado de satisfacción de 0 (nada satisfecho) a 10 (muy satisfecho). En el caso de haber recibido más de un formato de intervención psicológica, añádalo en la tabla.**

| **Formato** |  | **Satisfacción**  (de 0 a 10) |
| --- | --- | --- |
| **Individual** |  |  |
| **Grupal** |  |  |
| **Por Internet** (ordenador o móvil) |  |  |

**En caso de necesitar un tratamiento psicológico, ¿De qué manera preferiría recibirlo?** Marque el orden de preferencia: 1: el que preferiría en primer término; 2: el que preferiría en segundo lugar y 3: el que preferiría en tercer lugar. En caso de no desear recibir el tratamiento en alguna de estas categorías, dígalo.

| **MODALIDAD DE TRATAMIENTO** | **Orden** | **NO** |
| --- | --- | --- |
| ● ¿…de manera individual? |  |  |
| ● ¿…de manera grupal con personas con problemas similares al suyo? |  |  |
| ● ¿…a través de Internet (con una aplicación web en su ordenador o a través del teléfono móvil)? |  |  |

**Para finalizar, ¿Por qué ha elegido en primera opción ese formato (por ejemplo: individual)?**

**¿Por qué ha elegido en última opción ese formato (por ejemplo: a través de Internet)?**

**En caso de haber marcado alguna opción con NO, ¿Cuál es el motivo?**

**Muchas gracias por su participación en este estudio.**
