# Supplementary material for: What format of treatment do patients with emotional disorders prefer and why? Implications for public mental health settings and policies
Source: PLoS One. 2019 Jun 10;14(6):e0218117. doi: 10.1371/journal.pone.0218117 (PMC6557569; doi:10.1371/journal.pone.0218117)
Supplement: S2 File — English version (DOCX) [file pone.0218117.s002.docx]

**Patient's psychological format preferences survey**

(Jorge Osma, 2016. Universidad de Zaragoza)

**Center:**

**Main Diagnosis:**

**Identifying code of the user: Age: Gender:**

| Primary |  |
| --- | --- |
| Secondary |  |
| University |  |
| Higher |  |

**Marital status: Educational level:**

| Single |  |
| --- | --- |
| Married |  |
| In a relationship |  |
| Divorced |  |
| Widowed |  |

**Job status: Income (yearly):**

| Unemployed/no compensation |  |
| --- | --- |
| Unemployed/compensation |  |
| Temporary |  |
| Part-time |  |
| Full-time |  |

| 0-16.000 euros |  |
| --- | --- |
| 16.001-17.500 euros |  |
| 17.501-19.000 euros |  |
| 19.001-21.000 euros |  |
| 21.001- 24.000 euros |  |
| 24.001-66.451 euros |  |

| **Yes** |  | **No** |  |
| --- | --- | --- | --- |

**Have you previously received psychological treatment?** (It doesn’t matter if you have been uninterrupted or in different periods of life)

**If you have answered YES in the previous question, please answer the following questions:**

**What was the approximate number of sessions you have received?** (for example, 13 sessions).

|  |
| --- |

**Please indicate the format in which you received the treatments and their degree of satisfaction from 0 (not satisfied) to 10 (very satisfied). If you have received more than one format of psychological intervention you can add it in the table.**

| **Format** |  | **Satisfaction**  (From 0 to 10) |
| --- | --- | --- |
| **Individual** |  |  |
| **Group** |  |  |
| **Online**  (Computer or mobile) |  |  |

**In case you will need a psychological treatment, how would you prefer to receive it?** Choose the order of preference: 1: the one you would prefer first; 2: the one you would prefer second and 3: the one you would prefer third. If you do not wish to receive treatment in any of these categories, say no.

| **TREATMENT MODALITY** | **Order** | **NO** |
| --- | --- | --- |
| ● … in an individual way? |  |  |
| ● … in a group way with people with similar problems? |  |  |
| ● … online way (with a web application on your computer or mobile phone)? |  |  |

**Finally, Why did you choose that format in the first option? (for example: individual)?**

**Why did you choose that format in the third place? (for example: Online)?**

**If you have marked an option with NO, Why would you never choose this format?**

**Thank you very much for your collaboration in this study.**
